# Effects of Standard Physiotherapy with the Addition of Mechanical Traction on Pain, Physical Activity and Quality of Life in Patients with Knee Osteoarthritis

**DOI:** 10.3390/medicina61030507

**Published:** 2025-03-15

**Authors:** Kati Florjančič, Renata Vauhnik

**Affiliations:** 1 Department of Physiotherapy, Faculty of Health Sciences, University of Ljubljana, Zdravstvena pot 5, 1000 Ljubljana, Slovenia; kati.florjancic@hotmail.com; 2 Arthron, Institute for Joint and Sports Injuries, Ukmarjeva 2, 1000 Ljubljana, Slovenia

**Keywords:** physiotherapy, osteoarthritis, knee joint, traction

## Abstract

*Background and Objectives:* There is evidence of decreasing knee pain in patients with knee osteoarthritis when knee mechanical traction is performed surgically. Our aim was to measure the effects of standard physiotherapy with the addition of knee mechanical traction on pain, physical activity and quality of life in patients with knee osteoarthritis. *Materials and Methods:* A clinical observational study with intervention and without a control group was conducted at three outpatient health clinics on a primary level of the health care system. Twenty-three patients with knee osteoarthritis voluntarily participated in the study. Standard physiotherapy included education, therapeutic and aerobic exercise, conventional TENS, low-intensity laser and manual soft tissue techniques. Mechanical traction of 150 N continuous force for 15 min with the knee joint at 25° flexion was added to standard physiotherapy. The following outcome measures were used: VAS, Knee Injury and Osteoarthritis Outcome Score and a 30 s sit-to-stand test. *Results:* The pain measured for the VAS at rest (*p* < 0.001) and during movement (*p* < 0.001) as well as for the Knee Injury and Osteoarthritis Outcome Score pain part decreased (*p* < 0.05). The quality of life did not improve (*p* > 0.05), but the physical activity of the patients did (*p* < 0.05). A decrease in pain correlated with body mass (*p* < 0.05). *Conclusions:* Standard physiotherapy with the addition of mechanical traction had an effect on reducing pain and improving physical activity.

## 1. Introduction

Osteoarthritis (OA) is a chronic and degenerative joint disease known to cause the loss of articular cartilage, the formation of osteophytes and changes in the subchondral bone [1]. Oxidative stress in chondrocytes increases with age, increasing the likelihood of cartilage breakdown and altering mitochondrial function in cartilage tissue [2].

Currently, there is no drug to prevent the onset of osteoarthritis, so the disease is treated symptomatically with pain relief and lifestyle changes [3]. Effective physiotherapy techniques include education about the disease and risk factors, therapeutic exercise to improve muscle strength, aerobic exercise, transcutaneous electrical nerve stimulation (TENS), low-intensity lasers [4,5] and manual soft tissue techniques [6].

With knee joint distraction (KJD), the knee joint is surgically temporarily fully unloaded by the distraction of the tibia and femur for five millimeters over six weeks using an external fixation frame [7]. It has been proven to reduce pain and improve quality of life (QoL) in knee OA patients [8]. The mechanisms of KJD are not fully understood, but studies have shown an increase in collagen network synthesis [8], subchondral bone reorganization [9] and accelerated renewal of synovial fluid [10]. Patients can still fully bear weight and expose the knee to sufficient axial loads, which improves cartilage regeneration [11]. About half of the patients with KJD have extended the time to another knee surgery by up to nine years. However, an important disadvantage of KJD is the increased number of skin infections because the penetrated skin on the side of the insertion of external fixation is considered to be an open wound until its removal [7].

In physiotherapy, limb traction is less frequently used because its effects have yet to be studied. In the studies about knee traction in people with knee OA to date, authors have reported some methodological limitations. In addition, daily intervention lasting several weeks is often not possible.

Therefore, the aim of our study was to evaluate if standard physiotherapy with the addition of mechanical traction reduced pain and improved physical activity and quality of life in patients with knee OA.

## 2. Materials and Methods

This was a clinical observational study with intervention and no control group. The study was approved by the National Medical Ethics Committee of Slovenia (No. 0120-36/2021/3). Participants signed a written consent form before participation.

### 2.1. Inclusion and Exclusion Criteria

Patients from three outpatient health clinics who had previously been diagnosed with knee OA by a doctor and who were older than 30 were included in the study. Patients who (1) had been diagnosed with rheumatoid arthritis, (2) had drugs injected directly into the joint in the three months prior to the start of the study, (3) had received physiotherapy or taken steroidal or non-steroidal anti-inflammatory drugs 15 days before the start of the study, (4) had other pain in the lower limb (e.g., due to polyneuropathy, restless legs syndrome, fibromyalgia, hip pathology, nerve root compression, central nervous system disease, etc.) were excluded from the study.

### 2.2. Outcome Measurements

Measurements were taken before the first standard physiotherapy with the addition of mechanical traction and immediately after the eight interventions. First, a form about the patient’s demographic data was completed (side treated, age, sex, body mass, height, presence of menstruation or menopause, previous injuries and surgeries to the treated knee and previous lower limb surgeries) and an assessment of physical activity was conducted using the Tegner Activity Score. The Tegner Activity Score is a self-assessment questionnaire that provides an objective measurement of an individual’s level of physical activity. The levels of the scale are predefined, with level 0 representing sick leave or incapacity to work and level 10 indicating participation in competitive sport at a national elite level [12].

Pain intensity at rest and in motion was assessed using a visual analog scale (VAS) on a 10 cm line with written instructions for the patient. On the left side was the number zero, which, as written, indicated no pain. On the right-hand side was the number 100, which represented the worst imaginable pain. Patients were asked to use a pencil to mark the line corresponding with the intensity of pain they felt at rest and during movement. A VAS is a reliable measure of pain intensity for a population of patients with knee OA, with a small minimal detectable change (MDC = 0.08) [13] and a minimal clinically important difference (MCID) of 19.9 mm [14].

Pain and QoL were assessed using two subscales of the Slovenian translation of the Knee Injury and Osteoarthritis Outcome Score (KOOS) [15]. Patients were given verbal and written instructions to check the box under the statement with which they most agreed. Each question was later rated from zero to four (from left to right) on the form (Example: P1 How often does your knee hurt: (0) Never, (1) Monthly, (2) Weekly, (3) Daily, (4) Always). The maximal total score for the nine questions marked P1 to P9 about the intensity of the pain was 36 points. To convert the result into a scale from 0 to 100, we used the following formula: P = 100 − (sum of points from P1 to P9 × 100)/36. To evaluate the QoL, the maximal total score for the four questions marked Q1 to Q4 was 16 points. To convert the result to a scale from 0 to 100, we used the following formula: P = 100 − (sum of points from Q1 to Q4 × 100)/16. A score of 0 points indicated severe problems and 100 points indicated an absence of problems with the knee [16]. KOOS subscales for pain and QoL are internally consistent [17] and highly reliable [18] for patients with knee OA.

Physical activity was evaluated using the 30 s sit-to-stand test (STS30) [19] because it is a reliable performance test for evaluating physical activity in patients with knee OA [20], with an MDC of 1.27 sit-to-stands and an MCID of 2–3 sit-to-stands [21]. The test was performed using a standard chair (43 cm) without armrests, which was placed against a wall. The starting position of the patients was seated, with the back against the backrest, feet flat on the floor and arms crossed on the chest. We demonstrated the test procedure slowly and quickly. Patients were asked to perform as many as they could and had one attempt. The test began with the request ‘Now!’. A correct stand was counted when the patient stood upright and then sat back down in the starting position. The last rise from the chair was considered valid if the patient had at least reached an upright position. The result of the test was the number of complete rises within 30 s. To ensure blinding, the examiner silently counted the correctly completed stand-up attempts and did not report the remaining time on the clock [19].

### 2.3. Standard Physiotherapy

The intervention was applied eight times because this is the standard number of therapies for knee OA patients in Slovenia. Standard physiotherapy included education about the disease and risk factors, therapeutic exercise to improve the muscle strength of the lower limb and trunk, aerobic exercise, conventional TENS, low-intensity lasers for knee pain and manual soft tissue techniques. Details are described in the Supplementary Materials.

### 2.4. Mechanical Traction

Mechanical traction was applied after every standard physiotherapy session, either using an Eltrac 471 (Enraf-nonius, Rotterdam, The Netherlands) or a BTL 6000 Tracton (BTL Medical Technologies S.R.O., Prague, Czech Republic).

**Figure 1:**
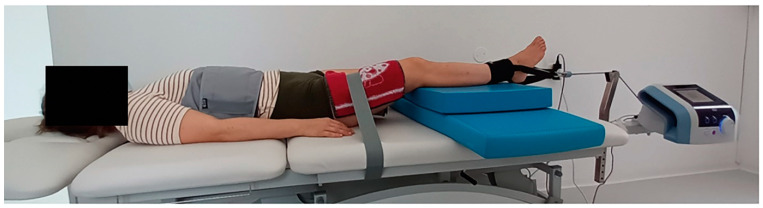
Placement of the body and the knee for mechanical traction.

Mechanical traction was applied with 150 N (or 15 kg) force because, in KJD, the same force used to achieve five millimeters of knee distraction has been proven to improve the symptoms of knee OA. Palhais et al. [22] also achieved the same distraction with mechanical traction at the same amount of force. The force was continuously applied for 15 min because the same application in other studies did not cause adverse events [23]. The knee joint was placed at 25° flexion, where joint structures are the most relaxed and the maximum amount of joint play is enabled. We achieved this by lying firm mats under the shin (Figure 1). Before starting, the angle was measured using a goniometer (Saehan Corporation, Seoul, Republic of Korea). Based on the results of the previously mentioned studies, we applied knee traction with a force of 150 N continuously for 15 min at 25° knee flexion on 8 consecutive working days.

### 2.5. Statistical Analysis

The statistical analysis was performed using R Commander 2.6-0 (Great Britain GNU General Public License) with the alpha level set at 0.05. Descriptive statistics were calculated at the baseline. We first used graphic methods to determine the type of distribution, such as the Q-Q plot and histograms. The Shapiro–Wilk test for normality was used to analyze the normal distribution of measured variables (*p*  >  0.5).

To determine if there was any change between before and after results on the mean scores of the intensity of pain at rest (VAS-R) and movement (VAS-M), pain detected using KOOS (KOOS-P), quality of life detected using KOOS (KOOS-QoL) and STS30, a paired Student’s *t*-test was used for normally distributed variables. The Wilcoxon signed-rank test was used if the distribution of variables was uneven. Mean differences and the 95% confidence interval were calculated.

To evaluate statistical power, we calculated the effect size (ES). We used Hedges’ *g* with the alpha level set at 0.05. We considered an ES up to 0.2 to be small (due to chance), from 0.2 to 0.5 to be moderate and above 0.8 to be large.

Multiple and simple linear regressions were used to test the correlation between VAS-R, sex and weight. Data were screened for normality assumptions, homogeneity of variance and the presence of extreme scores. In addition, the variance inflation factor (VIF) was calculated. We considered multicollinearity to be unproblematic for VIF values that were below 10.

For a statistically significant model, we calculated the 95% confidence interval for the independent variable, the adjusted coefficient of determination (R^2^) and Pearson’s *r*. We considered Pearson’s *r* to not indicate a correlation for values up to 0.25 (−0.25); the correlation for values between 0.25 and 0.50 (−0.25 and −0.50) was low, between 0.50 and 0.75 (−0.50 and −0.75) was moderate to high and above 0.75 (−0.75) was very high to excellent.

## 3. Results

Twenty-three subjects with knee OA aged 46 to 88 met the inclusion criteria. The descriptive statistics of the patients’ characteristics are presented in Table 1. There were no dropouts and none of the participants reported any adverse events. Some reported mild discomfort, but not pain.

The measured differences in VAS-R, VAS-M, KOOS-P and KOOS-QoL were normally distributed and a difference for STS30 was not present. The findings of the Shapiro–Wilk test are shown in Table 2.

A statistically significant decrease in pain intensity was measured for VAS and KOOS and we found large ESs for both measurement tools (ES_VAL_ = 0.96; ES_KOOS-P_ = 0.82). On the contrary, the measured change in the quality of life was not statistically significant, with a small ES (ES_KOOS-QoL_ = 0.19). However, the measured change in activity was statistically significant, with a moderate ES (ES_STS30_ = 0.77).

When creating a linear model for correlations between the VAS-M difference, sex and body mass, we found the need to check the interaction between the independent variables for sex and body mass. Due to the small sample size, we were aware that the trend might not be apparent; however, we were interested if a correlation existed between VAS-M difference, sex and body mass, even in a small number of patients.

By analyzing the variability of the residuals, we did not detect any violations in the assumptions of the linear model. The VIF did not indicate a problem with the model due to collinearity (VIF_sex_ = 1.05; VIF_body mass_= 1.05).

We did not find the correlation of sex to be statistically significant in the first multiple linear regression model. Therefore, we checked if the interaction between sex and body mass influenced the linear model. Further, we did not find the interaction statistically significant, although we removed sex and the interaction between sex and body mass from the linear model. Finally, we found the simple linear regression model to be statistically significant. Details of the statistical analysis are presented in Table 3.

We calculated the 95% confidence interval for body mass (95% CI = [0.14–6.97]), adjusted coefficient of determination (R^2^ = 0.1425) and Pearson’s correlation coefficient (*r* = 0.42).

## 4. Discussion

Twenty-three subjects with knee OA were included in the study to assess if standard physiotherapy with added mechanical traction of the knee reduced pain and improved activity and QoL in patients treated at Slovenian outpatient health clinics, thus assessing the change in variables over time. Our results showed that standard physiotherapy with the addition of mechanical traction reduced pain in knee OA patients and improved patients’ physical activity. Mechanical traction was applied with 150 N force because the same force is used in knee joint distraction to achieve five millimeters of knee distraction and has been proven to improve the symptoms of knee OA.

Our study’s findings about pain intensity aligned with the results of previous studies [24,25], even though different standard physiotherapy methods were used. Pain intensity for the VAS at rest decreased by 2.2 cm on average, while, during movement, it was 2.4 cm. The pain intensity during movement before therapy was, on average, higher than at rest, allowing more room for improvement. The statistical power of the results was high, indicating little variability in the results. In our study, we also exceeded the value of the smallest MCID reported, 1.99 cm [14]. Choi and Lee [24] applied intervention for four weeks, five times a week, with a decrease in pain of 4.73 cm on average for the VAS. Our study found that pain intensity could be reduced with just eight interventions. However, the results of Choi and Lee [24] suggest that a higher number of interventions may have a more significant effect on pain intensity. As a result, applying isolated traction force to the knee is challenging because it also affects the proximal joint, i.e., the hip joint. Also, Abdel-Aal et al. [25] reported that standard physiotherapy with the addition of continuous knee mechanical traction at 20° and 90° was more effective at reducing pain than the position at knee extension. The main difference between our study and the two mentioned studies is their use of paracetamol before applying mechanical traction. As the drug has a significant systemic effect on the central nervous system [26] and as knee OA patients may also show signs of central sensitization, the results measured immediately after therapy in these two studies may have been due to the use of paracetamol and not the therapy itself. In future studies, we recommend not using painkillers during the time of interventions as they may influence the results.

In our study, the pain intensity measured using KOOS-P also significantly decreased. The score increased by 8.2 points on average and, despite the small sample size, the effect size was large. The findings were consistent with the results measured using the VAS. If, on the VAS, the pain was reduced only at rest or during movement, we would not have expected a statistically significant improvement in the result for KOOS-P either because the questions for KOOS-P referred to knee pain both at rest and during movement. In one study, the authors also used the KOOS-P score to measure changes in pain intensity before and after standard physiotherapy with the addition of mechanical traction [27]. They reported a statistically significant score increase after ten interventions that remained statistically significant one month after treatment, indicating long-term effects. In their study, similar mechanical traction parameters to our study were used. However, unlike us, they also used surface heating and ultrasound as part of standard physiotherapy, which could have had an effect on reducing pain intensity. In other previous studies, the authors mostly used the WOMAC index to assess pain, stiffness and function, but the results of the index were presented as a total score as opposed to individual parts [22,24] and the findings considerably differed. The WOMAC index consists of three parts, so an interpretation of an individual part with the overall result is, therefore, not possible. The WOMAC index score can also be influenced by pain in other parts of the body as the questions refer to more generalized activities of daily living [28]. Alpayci et al. [23] are the only authors who have reported the results of individual parts of the WOMAC index and the total score of the WOMAC index. Their results support the findings of our research because the results of both studies showed a reduction in pain intensity and a trend towards improvements in the physical activity of the patients.

We measured QoL using the subscale of the KOOS questionnaire. The KOOS-QoL score increased by 3.3 points on average, which did not indicate an improvement in patients’ QoL. The ES was small or random, which aligned with the statistically insignificant results. Although the validity of the KOOS was confirmed in patients with knee AO who were awaiting surgery [29], our results suggest that eight interventions in three weeks were too few to detect significant changes in QoL. We decided to use the KOOS questionnaire because the measurement properties had been investigated in the given population. Using Cohen’s d, we calculated that the sample size should include 212 patients in order to reach a power of 0.8 for the KOOS-QoL score, confirming the above assumption.

Physical activity was measured using STS30. Even though the improvement was statistically significant by two sit-to-stands on average, a large effect size of 0.8 was not achieved (ES_STS30_ = 0.77). In order to reach a large effect size for the STS30 results, the sample should have included 32 patients. However, we reached the lower limit of the MCID reported by Wright et al. [21]. The results of our study using STS30 reflected a trend of improvement in physical activity, which can affect QoL in the long run.

To our knowledge, this is the only study to date that also includes a functional test to measure the change in the physical activity of patients with knee OA after standard physiotherapy with added mechanical traction. Khademi-Kalantari et al. [27] measured changes in mobility using a 6 min walking test.

**Figure 2:**
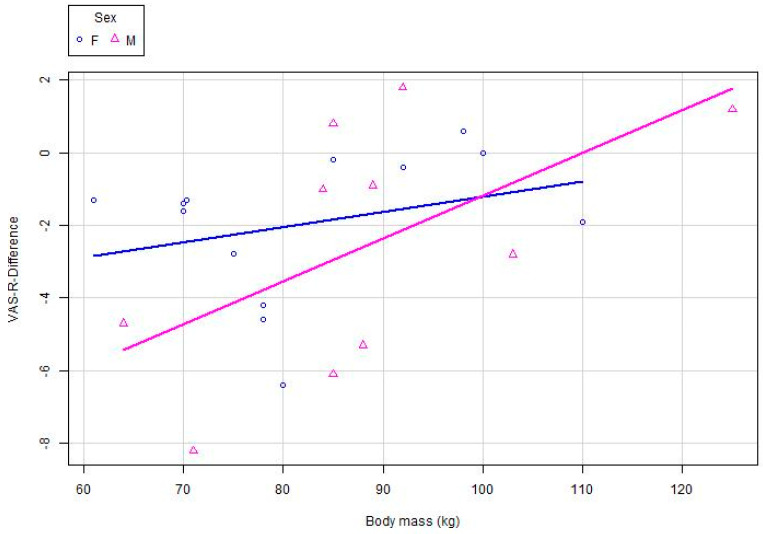
Linear correlation between the VAS-M difference and body mass in females and males.

Using a multiple linear regression model, we assessed the correlation between VAS-M difference, sex and body mass after standard physiotherapy with added mechanical traction. We found that the VAS-M difference did not depend on sex and on the interaction of sex with body mass. These findings could be of great value for future studies on the effect of standard physiotherapy with mechanical traction on pain at rest in patients with knee OA as the findings suggest that a sample that is not balanced by sex will not have a statistically significant effect on pain at rest. We also found a statistically significant low positive linear correlation between the VAS-M difference and body mass (Pearson’s *r* = 0.42), meaning that the differences were displayed as negative values (example: subject X had VAS-M_before_ = 5, VAS-M_after_ = 2 and VAS-M_difference_ = −3) (Figure 2).

Therefore, patients with knee OA in our study who had a higher body mass consequently had less pain reduction at rest than those with a lower body mass. When using a single linear regression model, body mass explained 14% of the variability in VAS-M differences. Thus, according to the results of our study, the effect of standard physiotherapy with the addition of mechanical traction on pain intensity at rest is more significant in patients with a lower body weight. Excess body mass is one of the leading risk factors for knee OA, and people with knee OA usually have difficulty losing body mass. Consequently, it is necessary to include education about the importance of physical activity and body mass loss when treating a patient with knee OA. If patients lost body mass before standard physiotherapy with added mechanical traction, they would reduce the influence of obesity as a risk factor and simultaneously have a higher probability of pain reduction.

The biggest limitation of our study was the small sample size due to the strict exclusion criteria and no follow-up measurements. For future studies, we recommend the use of more sensitive measuring tools to assess changes in QoL and follow-up measurements to determine long-term effects.

## 5. Conclusions

A combination of standard physiotherapy and mechanical traction had an effect on reducing pain and improving physical activity. The effects of mechanical traction alone were not investigated in the study, but it showed no adverse effects in patients with knee osteoarthritis and could be beneficial in the treatment of the pathology. The positive effect of standard physiotherapy with additional mechanical traction was lower in patients with a higher body mass.

## Figures and Tables

**Table 1:** Demographic characteristics of the patients before the study.

Variable	
Affected knee side (right/left)	9 (39.1%)/14 (60.9%)
Sex (M/F)	10 (43.5%)/13 (56.5%)
Menstruation/menopause	1 (7.7%)/12 (92.3%)
Age (years)	65.9 ± 9.5
M/F	59.7 ± 7.6/70.6 ± 8.7
Height (cm)	168.2 ± 9.5
M/F	176.8 ± 5.7/161.5 ± 5.6
Body mass (kg)	84.9 ± 15.3
M/F	88.6 ± 16.7/82.1 ± 14.2
Tegner	
Level 1	1 (4.3%) (1 F)
Level 2	10 (43.5%) (7 F; 3 M)
Level 3	6 (26.1%) (5 F; 1 M)
Level 4	4 (17.4%) (4 M)
Level 5	2 (8.7%) (2 M)
Injury to the affected knee (M/F)	4/3—trauma and dislocations
Surgery to the affected knee (M/F)	4/2—arthroscopic surgery; post-trauma surgery
Surgery to both lower limbs (M/F)	0/5—collateral knee or ankle surgery

M: male; F: female; cm: centimeter; kg: kilogram.

**Table 2:** Measurements before and after intervention, mean differences, distributions, effects after the intervention and Type II error estimations.

Variable	$\bar{x}$ ± SD	$\bar{x}$_diff_. ± SD	Shapiro–Wilk	Paired Student’s *t*	Hedges *g*	ES
VAS-R_before_ (cm)	5.2 ± 2.8	−2.2 ± 2.7	0.95 (*p* = 0.29) †	3.95 ** [1.05–3.36]	0.8	0.96
VAS-R_after_ (cm)	3 ± 2.4					
VAS-M_before_ (cm)	6.2 ± 2.3	−2.4 ± 2.6	0.93 (*p* = 0.12) †	4.37 ** [1.25–3.51]	0.8	0.96
VAS-M_after_ (cm)	3.9 ± 2.3					
KOOS-P_befor_ (points)	49.6 ± 13.3	8.2 ± 15.4	0.93 (*p* = 0.12) †	−2.55 * [(−14.85)–(−1.52)]	0.63	0.82
KOOS-P_after_ (points)	57.8 ± 11.9					
KOOS-QoL_before_ (points)	38.9 ± 14.4	3.3 ± 15.3	0.96 (*p* = 0.52) †	−1.02 ^NS^ [(−9.87)–3.37]	0.23	0.19
KOOS-QoL_after_ (points)	42.1 ± 12.4					
STS30_before_ (num.)	9.5 ± 3	2 ± 1.1	0.89 (*p* = 0.01)	0 **	0.59	0.77
STS30_after_ (num.)	11.4 ± 3.3					

$\bar{x}$: mean; $\bar{x}$_diff_.: mean difference; ± SD: standard deviation; ES: effect size; VAS: visual analog scale; R: rest; M: movement; cm: centimeter. *p*-Value indicates statistical significance: * *p* < 0.05; ** *p* < 0.001; † normal distribution. KOOS: Knee injury and Osteoarthritis Outcome Score; QoL: quality of life; ^NS^: no statistical difference; STS30: 30 s sit-to-stand; num.: number.

**Table 3:** Statistical analysis of the multiple and single linear regression models.

	Estimate	SE	*t* Value	*p*-Value
Intercept	−9.64335	3.23752	−2.979	0.00742 *
Sex	1.08106	1.07188	1.009	0.32523
Body mass	0.08040	0.03544	2.268	0.03454 *
With interaction [sex x body mass]				
Intercept	−12.98063	4.45440	−2.914	0.0089 *
Sex	7.61621	6.11448	1.246	0.2281
Body mass	0.11807	0.04949	2.386	0.0276 *
Sex x body mass	−0.07662	0.07059	−1.085	0.2913
Without interaction [sex x body mass] and body mass				
Intercept	−8.37977	2.98650	−2.806	0.0106 *
Body mass	0.07272	0.03463	2.100	0.0480 *

SE: standard error; x: interaction; * statistically significant.

## Data Availability

The raw data supporting the conclusions of this article will be made available by the authors on request.

## Supplementary Materials

The following supporting information can be downloaded at: https://www.mdpi.com/article/10.3390/medicina61030507/s1. Questionnaire KOOS.
