# Supplementary material for: Effects of Standard Physiotherapy with the Addition of Mechanical Traction on Pain, Physical Activity and Quality of Life in Patients with Knee Osteoarthritis
Source: Medicina (Kaunas). 2025 Mar 15;61(3):507. doi: 10.3390/medicina61030507 (PMC11943517; doi:10.3390/medicina61030507)
Supplement: Supplementary file 1 [file medicina-61-00507-s001.zip › medicina-3500591-supplementary.pdf]

KOOS – VPRAŠALNIK O TEŽAVAH S KOLENSKIM SKLEPOM

Datum: _____/______/______

Številka preiskovanca: ____________________________________

NAVODILA: S tem vprašalnikom želimo izvedeti vaše mnenje o vašem kolenu. Pridobljeni podatki nam bodo pomagali spremljati ob**č**utke o vašem kolenu in to, kako dobro zmorete opravljati vaše obi**č**ajne aktivnosti.

Na vprašanje odgovorite tako, da ozna**č**ite okvir**č**ek pri ustreznem odgovoru. Pri vsakem vprašanju lahko ozna**č**ite samo <u>en</u> okvir**č**ek. **Č**e niste prepri**č**ani o svojem odgovoru, izberite odgovor, ki se vam zdi najustreznejši.

Bolečina

P1. Kako pogosto ob**č**utite bole**č**ino v kolenu?

Nikoli	Najmanj enkrat na mesec	Najmanj enkrat na teden	na dan	Vedno
☐	☐	☐	☐	☐

Kako mo**č**na je bila v **preteklem tednu** bole**č**ina pri opravljanju naslednjih aktivnosti?

P2. Pri obra**č**anju telesa/vrtenju na kolenu

Je ni bilo	Blaga	Zmerna	Huda	Zelo huda
☐	☐	☐	☐	☐

P3. Pri popolnemu iztegovanju kolena

Je ni bilo	Blaga	Zmerna	Huda	Zelo huda
☐	☐	☐	☐	☐

P4. Pri popolnemu pokr**č**enju kolena

Je ni bilo	Blaga	Zmerna	Huda	Zelo huda
☐	☐	☐	☐	☐

P5. Pri hoji po ravni površini

Je ni bilo	Blaga	Zmerna	Huda	Zelo huda
☐	☐	☐	☐	☐

P6. Pri hoji po stopnicah navzgor ali navzdol

Je ni bilo	Blaga	Zmerna	Huda	Zelo huda
☐	☐	☐	☐	☐

P7. Ponoči v postelji

Je ni bilo	Blaga	Zmerna	Huda	Zelo huda
☐	☐	☐	☐	☐

P8. Pri ležanju ali sedenju

Je ni bilo	Blaga	Zmerna	Huda	Zelo huda
☐	☐	☐	☐	☐

P9. Pri stanju pokonci

Je ni bilo	Blaga	Zmerna	Huda	Zelo huda
☐	☐	☐	☐	☐

Kvaliteta življenja

Q1. Kako pogosto se zavedate težav s kolenom?

Nikoli	Najmanj enkrat na mesec	Najmanj enkrat na teden	Najmanj enkrat na dan	Vedno
☐	☐	☐	☐	☐

Q2. Ali ste spremenili način življenja zato, da bi se izognili morebitnim aktivnostim, ki so škodljive za vaše koleno?

Sploh ne	Malo	Zmerno	Močno	Popolnoma
☐	☐	☐	☐	☐

Q3. Kako močno vas skrbi pomanjkanje zaupanja v koleno?

Sploh ne	Malo	Zmerno	Močno	Zelo
☐	☐	☐	☐	☐

Q4. Na splošno, kako velike so vaše težave s kolenom?

Jih ni bilo	Blage	Zmerne	Hude	Zelo hude
☐	☐	☐	☐	☐
